# CRM197-conjugated peptides vaccine of HCMV pp65 and gH induce maturation of DC and effective viral-specific T cell responses

**DOI:** 10.1080/21505594.2023.2169488

**Published:** 2023-02-01

**Authors:** Shuyun Zhang, Fulong Nan, Shasha Jiang, Xiaoqiong Zhou, Delei Niu, Jun Li, Hui Wang, Xueming Zhang, Xianjuan Zhang, Bin Wang

**Affiliations:** Department of Pathogenic Biology, Department of Special Medicine, School of Basic Medicine, Qingdao University, Qingdao, China

**Keywords:** Human cytomegalovirus, peptide-CRM197 vaccine, antigen presentation, maturation of DCs, T cell immunity

## Abstract

Human cytomegalovirus (HCMV) infection is prevalent worldwide, and there is currently no licenced HCMV vaccine to control it. Therefore, developing an effective HCMV vaccine is a significant priority. Because of their excellent immunogenicity, the crucial components of HCMV, phosphoprotein 65 (pp65) and glycoproteins H (gH) are potential target proteins for HCMV vaccine design. In this study, we predicted and screened the dominant antigenic epitopes of B and T cells from pp65 and gH conjugated with the carrier protein cross-reacting material 197 (CRM197) to form three peptide-CRM197 vaccines (pp65-CRM197, gH-CRM197, and pp65-CRM197+gH-CRM197). Furthermore, the immunogenicity of the peptide-CRM197 vaccines and their effects on dendritic cells (DCs) were explored. The results showed that three peptide-CRM197 vaccines could induce maturation of DCs through the p38 MAPK signalling pathway and promote the release of proinflammatory factors, such as TNF-α and interleukin (IL) −6. Meanwhile, the peptide-CRM197 vaccines could effectively activate T cell and humoral immunity, which were far better than the inactivated HCMV vaccine. In conclusion, we constructed three peptide-CRM197 vaccines, which could induce multiple immune effects, providing a novel approach for HCMV vaccine design.

## Introduction

Human cytomegalovirus (HCMV) is a double-stranded DNA virus belonging to the herpesvirus that infects approximately 60% of adults in developed countries and more than 90% in developing countries. Similar to all herpesviruses, HCMV establishes latency and persists for the life of the individual [1]. HCMV is not highly contagious, there are usually no symptoms associated with HCMV infection in healthy people [2]. Primary HCMV infection and its reactivation in immunocompromised populations may cause retinitis, encephalitis, neonatal mental retardation, and glioma [3,4]. HCMV infection and mortality are higher in patients with acquired immunodeficiency syndrome, transplant recipients, and developing foetuses [3], leading to a substantial social burden. There are currently have drugs to provide a major advance in HCMV disease management, but they suffer from limited effects, significant toxicity, and have side effects after long-term use [5]. Therefore, developing an effective HCMV vaccine is a priority project for preventing HCMV-related diseases [6]. Several HCMV vaccines candidates have been developed, including first-generation vaccines (live-attenuated Towne vaccine), second-generation vaccines (gB protein-based peptide vaccines, virus-like particles), and third-generation vaccines (nucleic acid vaccines) [7]. When these vaccines are injected in the body, they can activate lymphocytes to kill the infected cell and antigen-presenting cells (APCs), such as dendritic cells (DCs), macrophages, and monocytes. However, there is still no HCMV vaccine available for clinical. It is obvious that the development of HCMV vaccines to produce effective antibodies and immunocytes has still a long way to go [8].

The HCMV genome encodes more than 200 proteins that play significant roles in HCMV infection, such as immune evasion, viral DNA release, and viral replication cycle regulation [9,10]. The envelope proteins of HCMV are a possible target for developing an effective vaccine [9]. Compared with other envelope proteins, phosphoprotein 65 (pp65) is the most abundant tegument protein constituting the extracellular virion [9], which is involved in the suppression of both humoral and cellular immunity during HCMV infection [10]. As an essential target of HCMV-specific CTLs, pp65 epitopes with superior immunogenicity can be recognized by 70% − 90% of HCMV-specific CTLs [11]. In addition to pp65, glycoprotein H (gH) binds to the complexes of cell surface receptors, which is necessary for cell fusion and viral entry [12]. Additionally, gH is involved in initiating the transcription of viral genes and plays a crucial role in the replication cycle. Therefore, gH is also an essential target for the host immune response. Nevertheless, only few vaccines that target gH are available [13].

DCs are specialized APCs present in low numbers in the periphery but play a vital role in the immune system. DCs recognize antigens, and induce phagocytosis of antigens to generate MHC-peptide complexes, increasing the expression of costimulatory molecules CD80, CD86, and CD40 and enhancing the production of cytokines, which finally stimulate naive T cells to activate adaptive immunity [14,15]. Moreover, cytokines produced by DCs amplify the positive feedback signals provided by differentiated T cells [16,17]. Th1 cells secrete interferon-γ (IFN-γ), which is related to the clearance of intracellular microorganisms (primarily viruses). Th2 cells secrete specific interleukin (IL) proteins, such as IL-4, IL-5, and IL-13, to promote B cell proliferation and antibodies secretion [18]. The primary goal of vaccines and immunotherapy is to obtain CD8+T cells with superior qualitative characteristics which is critical for the initial step of the response [19]. Cross-Reacting Material 197 (CRM197) is a non-toxic mutant of diphtheria toxin (DT), with a mutation at position 52 [20], which reduces toxicity while retaining the same immunostimulatory properties as DT [21]. CRM197 activates CD4+T cells through heterogeneous Th1 and Th2 cytokine profiles to activate B cells and regulate antibodies [22]. Thus, CRM197 has been successfully used as a carrier in many vaccines for infectious diseases [23].

With the rapid development of bioinformatics, molecular biology-assisted software has been widely employed to predict protein epitopes in diagnostic reagent preparation and peptide vaccine synthesis [24]. Notably, using bioinformatics to analyse protein structure and predict antigen epitopes is more efficient than traditional experimental methods [25]. Immunoinformatic approaches have been proposed by several research groups against infectious diseases, such as Herpes Simplex virus [26] Hepatitis C virus [27], Zika virus [28], Human Immunodeficiency virus [23]. Thus, in this study, we predicted and screened the superior B and T cell epitope sequences in pp65 and gH of HCMV to couple with the carrier protein CRM197 as the peptide-CRM197 vaccines (pp65-CRM197, gH-CRM197, and pp65-CRM197+gH-CRM197). In addition, we explored the mechanism of peptide-CRM197 vaccine induced maturation of DCs *in vitro* and *in vivo*. Since HCMV lacks suitable animal models, so we further evaluated the immunogenicity of the peptide-CRM197 vaccines in mice, which provided a novel and reliable design for the HCMV vaccine.

## Materials and methods

### Screening of protein sequences

We analysed pp65 and gH of AD169 strain for antigenic epitope prediction. T and B cell epitopes located at β-turns and random coils were analysed using the SOPMA (http://npsa-pbil.ibcp.fr/cgi-bin/npsa_automat.pl?page=npsa_sopma.html) and IEDB (https://www.iedb.org/) databases [29]. T cell epitopes of gH and pp65 were predicted using IEDB and SYFPEITHI (http://www.syfpeithi.de/) [30], and the alleles predicted based on CD4+T epitopes were selected from HLA-DRB1. CD4+T antigenic epitopes with adjusted rank ≤1 in IEDB and score >20 in SYFPEITHI were selected. In the prediction of CD8+T epitopes, antigenic epitopes with consensus score ≤2 in IEDB and score >20 in SYFPEITHI were selected [31,32]. We employed the Bepipred Linear Epitope Prediction 2.0 method in IEDB to predict B cell epitopes, keeping all parameters set to default [33]. Overlapping epitopes of CD4+T and CD8+T cells with the highest scores in the two software were screened.

### Peptide synthesis

The overlapping epitopes of T and B cell with the highest scores were screened. Peptides were synthesized by Sangon Biotech, China and identified by high performance liquid chromatography and mass spectrometry with a purity >90 %. Then the peptides were dissolved in PBS and stored at −20 ℃.

### Preparation of conjugated peptides

**Figure 1.**
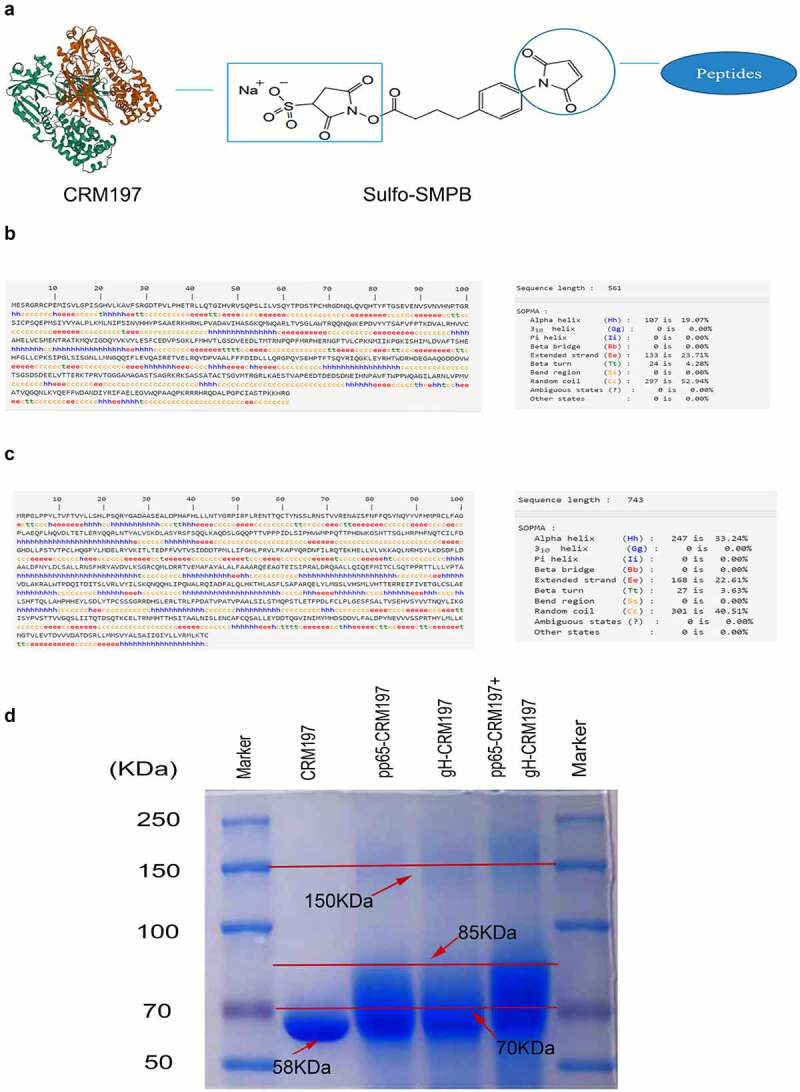
Protein structure prediction of pp65 and gH and preparation of coupled peptide vaccines. (a) the coupled diagram of peptide-CRM197. The secondary structure of pp65 (b) and gH (c) were predicted by the online software SOPMA. (d) After coupling the peptides with CRM197, three peptide-CRM197 were subjected to Coomassie brilliant blue staining after SDS-PAGE.

The C-terminal sulfhydrylated pp65 and gH peptides were dissolved in double-distilled water. CRM197 was dissolved in PBS to obtain 0.1 mM solution and incubated with 20-fold molar excess sulpho-SMPB (Thermo Fisher Scientific) at room temperature for 30 min. Subsequently, the SMPB/CRM197 mixture was desalted on the desalination column. Then, the activated CRM197 was incubated with pp65 and gH peptides at room temperature for 30 min (CRM197: polypeptide = 1:60). Then desalted on the desalination column. The coupled method is shown in Figure 1a. The coupled results were confirmed by SDS-PAGE and Coomassie staining.

### Cell cultures and virus

DC2.4 cells and HELF cells were purchased from the Shanghai Cell Resource Centre of the Chinese Academy of Sciences. The cells were cultured in DMEM medium supplemented with 10% foetal bovine serum (FBS) and 100 U/mL penicillin-streptomycin. The laboratory HCMV strain AD169 was proliferated in HELF cells. The virus was stored at −80℃.

### Detection of DCs activation

DC2.4 cells were treated with three peptide-CRM197 vaccines for 48 h. Flow cytometry was used to detect cell surface markers. The cells were detected with FITC-CD11c, BV605-CD80, BV421-CD86, BV605-MHCII, and PE-CD40. The levels of cytokines (TNF-α, IL-6, IL-12p70, and IL-1β) were detected using ELISA kits purchased from Servicebio. The mice were immunized with CRM197, pp65-CRM197, gH-CRM197, pp65-CRM197 + gH-CRM197. After three days, the mice were sacrificed to measure the maturation of DCs.

### Western blotting

DC2.4 cells were cultured in 6-well-plate at 1 × 10^6^/well overnight. Then treated with three peptide-CRM197 vaccines (2 ug/mL). LPS was used as the positive control. After 4 h, DC2.4 cells were collected and lysed in RIPA buffer. The protein samples were separated by SDS-PAGE and transferred to a PVDF membrane. The membrane was blocked for 2 h at room temperature. Then incubated with primary antibodies overnight at 4℃. The next day, secondary antibodies were added and the membrane was incubated for 2 h at room temperature. ECL reagent was used to observe protein by chemiluminescence. The primary antibodies used in the experiment include p-MEK-3/6 (B-9) mouse monoclonal antibody (1:200, Santa Cruz Biotechnology), p-p38 MAPK (D-8) mouse monoclonal antibody (1:200, Santa Cruz Biotechnology), p38 MAPK rabbit mAb (1:1000, ABclonal), anti-MAP2K6 rabbit polyclonal antibody (1:1000, Sangon Biotech), and anti-β-tubulin antibody (1:1000, Boster). The secondary antibodies used in the experiment include HRP goat anti-mouse IgG (H+L) (1;8000, ABclonal), and HRP goat anti-rabbit IgG (H+L) (1:8000, ABclonal).

### Mice immunization

To test the protective and response of the coupled peptides, female Balb/C mice (6–8 weeks old) were obtained from SPF (Beijing) Biotechnology Co. Ltd. They were divided into seven groups (six mice/group): PBS, OVA, CRM197, pp65-CRM197, gH-CRM197, pp65-CRM197+gH-CRM197, and inactivated HCMV (AD169) groups. The test groups were vaccinated intramuscularly with 20 μg/mouse of each vaccine antigen. The first immunization on day 0 was followed by two boosters on days 14 and 28 of the same amounts of peptide-CRM197 vaccines or CRM197 [34,35]. On day 35, mice were sacrificed to collect spleens and serum for further research.

### Cellular immune response

On day 35, mice were sacrificed to measure the expression of proinflammatory cytokines. Spleens were removed aseptically to prepare the single-cell suspension. 1 × 10^6^ cells/well were seeded in 96-well-plate. Inactivated HCMV was used as the antigen to stimulate spleen lymphocytes. After 48 h, flow cytometry was used to analyse lymphocyte subsets. T lymphocyte subtypes were detected using PB450-CD3, BV605-CD4, and FITC-CD8; CD4+T lymphocytes were detected using PE-IFN-γ, APC-IL-2, and PE-IL-4; CD8+T lymphocytes were detected using PE/CY7-TNF-α, and PE-IFN-γ. Treg cells were defined as CD4+CD25+Foxp3+. All flow cytometry antibodies were purchased from BioLegend.

### Lymphocyte proliferation assay

The proliferation ability of three peptides-CRM197 vaccines on mice lymphocytes was evaluated using a CCK-8 kit (Meilunbio). On day 35, spleens were extracted to prepare lymphocyte single-cell suspension, which were cultured in 96-well plate at 1 × 10^6^/mL in RPMI 1640 medium containing 10% FCS, with a total volume of 100 µL. The 96-well plate was incubated after being restimulated with inactivated HCMV (MOI = 3) [36,37]. CCK8 solution (10 μL) was added into each well at 48 and 72 h. Then, the plates were continuously cultured at 37℃ for 2 h each time, Absorbance at 450 nm was measured with an enzyme mark instrument to detect cell proliferation.

### 2.10. Neutralization assay

1.5 × 10^4^ cells/well HELF cells were seeded in the 96-well plate (NEST). Complement of immunized serum was inactivated at 56℃ for 30 min. The diluted serum was incubated with HCMV at 37℃ for 2 h, then the serum-HCMV mixture (100 µL/well) was added to HELF cells and cultured for 4–7 days. The morphological changes in HELF cells were observed under the microscope. Neutralization titres were calculated using the Reed-Muench method.

### 2.11. Statistical analysis

All data were processed using GraphPad Prism 9.0. The results were expressed as mean ± SD (standard deviation). One-way analysis of variance (ANOVA) was used to compare the differences among the groups. Tukey’s multiple comparisons test was performed to compare the mean values among various groups. Data from different mice/wells, *P < .05, **P < .01, ***P < .001 and ****P < .0001, denoted statistical significance.

## Results

### Prediction of antigen epitopes and preparation of peptide vaccines

The secondary structures of pp65 (Figure 1b) and gH (Figure 1c) were analysed using SOPMA. B and T cell epitopes, which were located at β-turns and random coils, were predicted using IEDB and SYFPEITHI. There were 20 B cell epitopes, 12 CD4+T cell epitopes, and 46 CD8+T cell epitopes in pp65. Among all sequences, 5 B cell epitopes and 10 T cell epitopes with high scores that could be recognized by both CD4+T cells and CD8+T cells were selected (Table 1). VNVHNPTGRSICPSQE (92–107) being the most repetitive sequence, was chosen as the final peptide vaccine. In gH, 23 B cell epitopes, 11 CD4+T cell epitopes, and 38 CD8+T cell epitopes were obtained. Then, five B cell epitopes, and nine T cell epitopes with high scores that could be recognized by CD4+ and CD8+ T cells were selected (Table 2). The most repetitive sequence ARQEEAGT (353–360) was selected as the final peptide vaccine. The peptides were synthesized and coupled with CRM197, and then identified by Coomassie staining following SDS-PAGE. As shown in Figure 1d, CRM197 (Lane 2) exhibited a single band corresponding to 58 kd. Compared with CRM197, pp65-CRM197 (Lane 3), gH-CRM197 (Lane 4), and pp65-CRM197+gH-CRM197 (Lane 5) all showed a single electrophoretic band with slow mobility, corresponding to molecular weight of more than 70 kd, indicating that CRM197 was successfully coupled with the polypeptides.

**Table 1.** Prediction of pp65 epitopes.

Cell epitope	Restricted HLA allele	site	Epitope Sequence
CD8+T	HLA-A *01:01	101-112	SICPSQEPMSIY
	HLA-A *02:06	105-113	SQEPMSIYV
	HLA-A *31:01	90-100	VSVNVHNPTGR
	HLA-A *30:02	100-112	RSICPSQEPMSIY
	HLA-B * 07:02	101-111	SICPSQEPMSI
	HLA-B * 14:02	93-102	NVHNPTGRSI
	HLA-C * 04:01	94-102	VHNPTGRSI
CD4+T	HLA-DRB1 * 01:09	104-121	PSQEPMSIYVYALPLKML
	HLA-DRB1 * 01:13	105-121	SQEPMSIYVYALPLKML
	HLA-DRB1 * 01:03	104-121	PSQEPMSIYVYALPLKML
B		28-36	SRGDTPVLP
		61-75	YTPDSTPCHRGDNQL
		94-109	VHNPTGRSICPSQEPM
		169-181	TRQQNQWKEPDVY
		248-269	SDVEEDLTMTRNPQPFMRPHER

**Table 2.** Prediction of gH epitopes.

Cell epitope	Restricted HLA allele	site	Epitope Sequence
CD8+T	HLA-A *33:03	357-366	EAGTEISIPR
	HLA-A *33:03	345-354	YALALFAAAR
	HLA-B * 13:01	354-362	RQEEAGTEI
	HLA-B * 40:01	353-364	ARQEEAGTEISI
	HLA-C * 12:03	350-358	FAAARQEEA
CD4+T	HLA-DRB1 * 01:01	342-356	AFAYALALFAAARQE
	HLA-DRB1 * 01:20	341-357	MAFAYALALFAAARQEE
	HLA-DRB1 * 01:04	342-356	AFAYALALFAAARQE
	HLA-DRB1 * 01:26	343-357	FAYALALFAAARQEE
B		1-6	MRPGLP
		143-162	LKAQDSLGQQPTTVPPPIDL
		168-187	WMPPQTTPHDWKGSHTTSGL
		354-363	RQEEAGTEIS
		542-550	DATVPATVP

### Three peptide – CRM197 vaccines activated DCs in vitro

**Figure 2.**
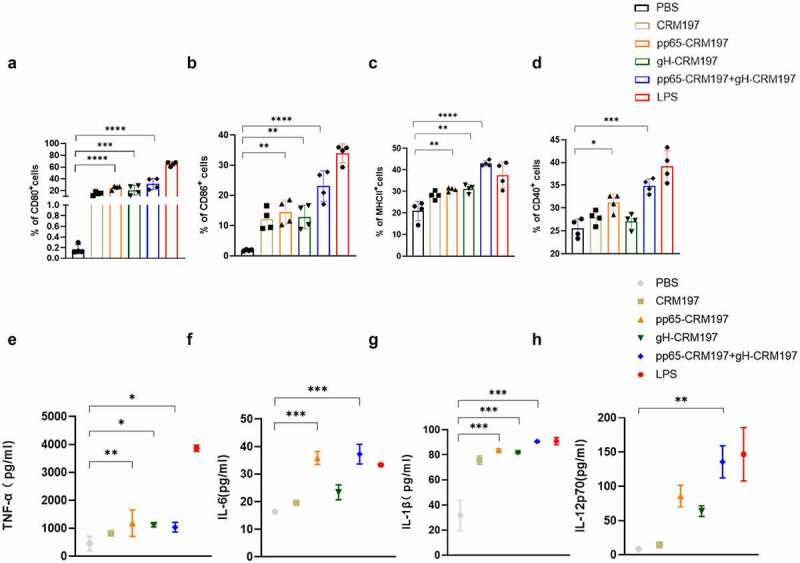
Analysis of three peptide-CRM197 vaccines for maturation of DCs *in vitro*. DC2.4 cells were stimulated with PBS, LPS, and three peptide-CRM197 vaccines for 48 h. The percentages of CD11c+ DCs expressing CD80 (a), CD86 (b), MHCII (c), and CD40 (d) were compared by flow cytometry. At the same time, the cell culture supernatants were harvested to detect the secretion levels of TNF-α (e), IL-6 (f), IL-1β (g), and IL-12p70 (h). All data were expressed as mean ± SD, * means *P* < .05, ** means *P* < .01, *** means *P* < .001, and **** means *P* < .0001 (one-way ANOVA).

To understand the mechanism underlying the promotion of DCs maturation by the peptide – CRM197 vaccines *in vitro*, we stimulated DC2.4 cells with three peptide – CRM197 vaccines respectively. We measured the expression of surface markers and proinflammatory cytokines using flow cytometry and ELISA. Compared with the PBS group, the expression of CD80, CD86, and MHCII in all peptide – CRM197 vaccines treated groups upregulated significantly (Figures 2a-c), especially the ratio of CD80 (Figure 2a) and CD86 (Figure 2b) in the pp65-CRM197+gH-CRM197 group reached 30%, while that of the PBS group was less than 5%. Surprisingly, the expression of MHCII (Figure 2c) and CD40 (Figure 2d) in the pp65-CRM197 +gH-CRM197 vaccine treated group was even higher than that in LPS treated group (positive control group). Correspondingly, all peptide – CRM197 vaccines induced higher levels of cytokines (TNF-α, IL-6, IL-1β, and IL-12p70), especially in the pp65-CRM197 + gH-CRM197 group (Figures 2e-h), the secretion of TNF-α reached 1200 pg/mL, which was much higher than that in the PBS group (300 pg/mL). These results indicated that three peptide – CRM197 vaccines activated DC2.4 cells, resulting in effective antigen presentation *in vitro*.

### Three peptide – CRM197 vaccines activated DCs in vivo

**Figure 3.**
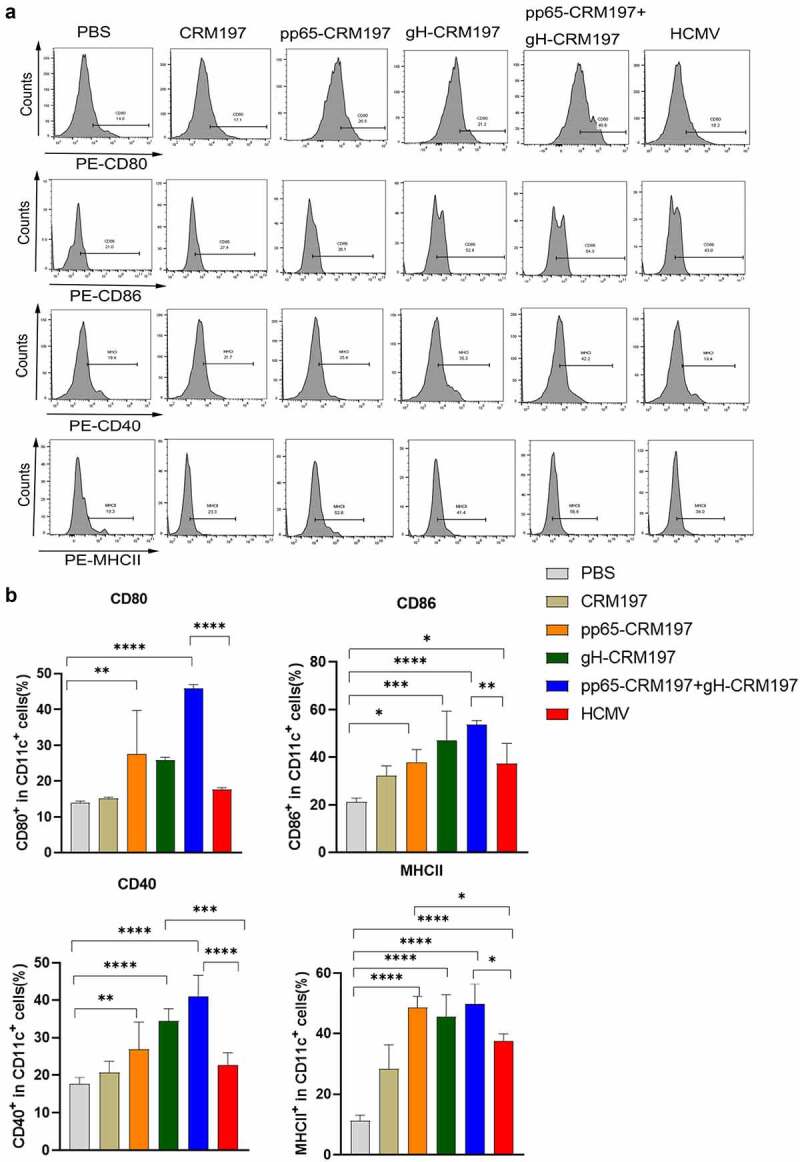
Analysis of three peptide-CRM197 vaccines for maturation of DCs *in vivo*. The mice were immunized with PBS, inactivated HCMV, and peptide-CRM197 vaccines. On the 3rd day, the expression of CD80, CD86, CD40, and MHCII in CD11c+ cells was analysed by flow cytometry. (a) the histograms of flow cytometric analyses of CD80, CD86, CD40, and MHCII in CD11c+ cells. (b) the percentages of CD11c+ DCs expressing CD80, CD86, CD40, and MHCII. All data were expressed as mean ± SD, * means *P* < .05, ** means *P* < .01, *** means *P* < .001, and **** means *P* < .0001 (one-way ANOVA).

To examine the initiation of an innate immune response by three peptide – CRM197 vaccines *in vivo*, the mice were immunized with CRM197, pp65-CRM197, gH-CRM197, pp65-CRM197+gH-CRM197, and inactivated HCMV. Three days after immunization, the expression of surface markers of DCs in spleens was detected by flow cytometry. Figures 3a-b indicated that three peptide – CRM197 vaccines increased CD80, CD86, CD40, and MHCII expression in CD11c+ cells compared with the PBS group. Noteworthily, the pp65-CRM197+gH-CRM197 vaccine stimulated higher levels of surface markers expression in the CD11c+ cells than that in the inactivated HCMV group, especially the ratio of CD80 reached 50%, which was significantly higher than that in the inactivated HCMV group (20%). This finding indicated that three peptide – CRM197 vaccines could also activate DCs *in vivo*.

### Three peptide – CRM197 vaccines activated DC2.4 cells via the p38 MAPK pathway

**Figure 4.**
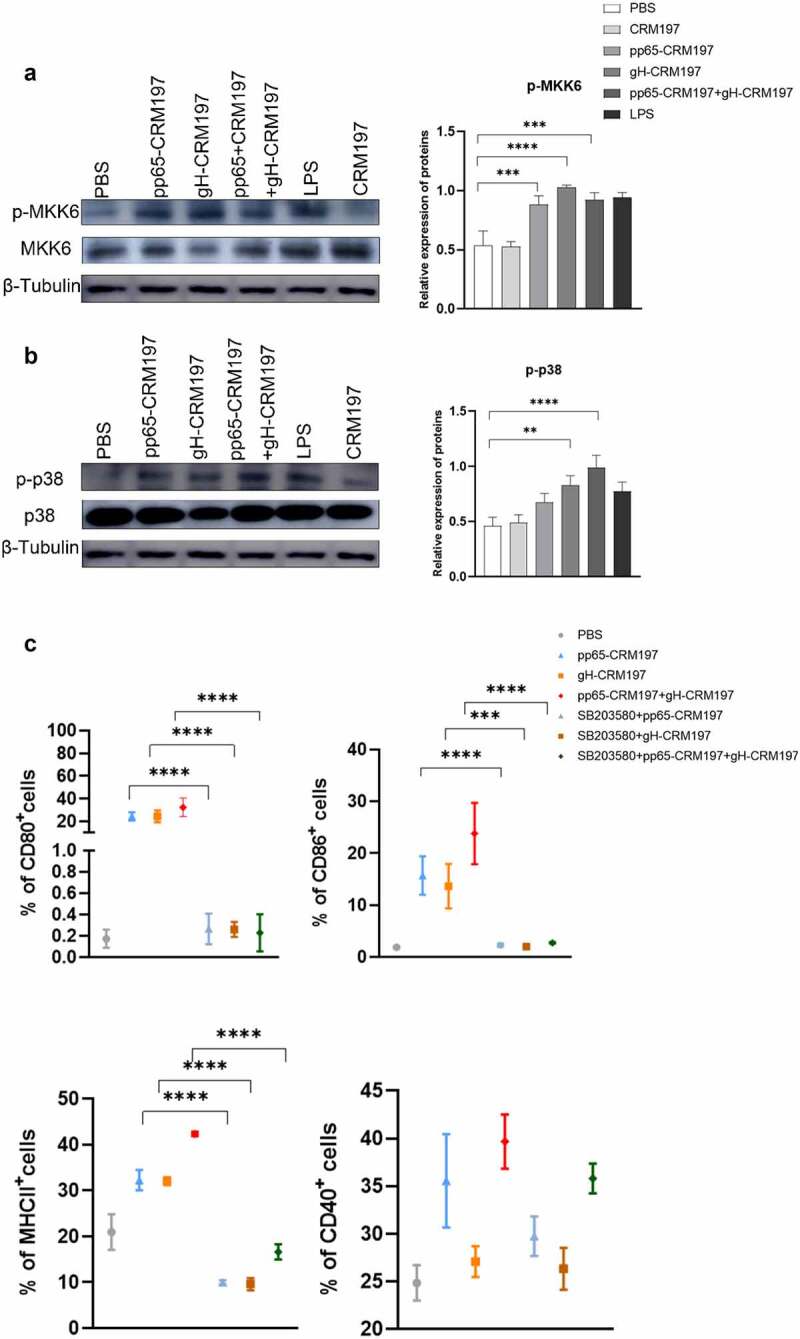
The signalling mechanism of activating DCs by three peptides-CRM197 vaccines. DC2.4 cells were stimulated by PBS, LPS, and peptide-CRM197 vaccines for 48 h, cellular proteins were extracted, and the expression of p-MKK6 (a) and p-p38 (b) were detected by Western blotting. (c) DC 2.4 cells were pretreated with SB203580 for 1 h, then were stimulated with PBS, LPS, and three peptide-CRM197 vaccines for 48 h. The expression of CD80, CD86, MHCII, and CD40 was detected by flow cytometry. All data were expressed as mean ± SD, * means *P* < .05, ** means *P* < .01, *** means *P* < .001, and **** means *P* < .0001 (one-way ANOVA).

To identify whether p38 MAPK activation was involved in peptide – CRM197 vaccine-mediated DCs maturation, we first analysed the activation of p38 MAPK signalling in DCs by Western blotting. The results indicated that the expression of essential proteins, such as p-p38 and p-MKK6 was significantly enhanced in DCs after treated with three peptide – CRM197 vaccines (Figures 4a-b). Then, DC2.4 cells were pretreated with SB203580 (an inhibitor of p38 MAPK signalling) for 1 h and stimulated with three peptide – CRM197 vaccines, respectively. The inhibition of p38 MAPK signalling significantly reduced the expression of CD80, CD86, CD40, and MHCII in all peptide – CRM197 vaccines stimulated DCs (Figures 4c and S1). The ratio of CD80 and MHCII decreased by approximately 30% after treatment with the inhibitor and that of CD86 decreased by 10%. Therefore, these results indicated that three peptide – CRM197 vaccines may activate DCs through the p38 MAPK signalling.

### Evaluation of the immunogenicity of three peptide – CRM197 vaccines

**Figure 5.**
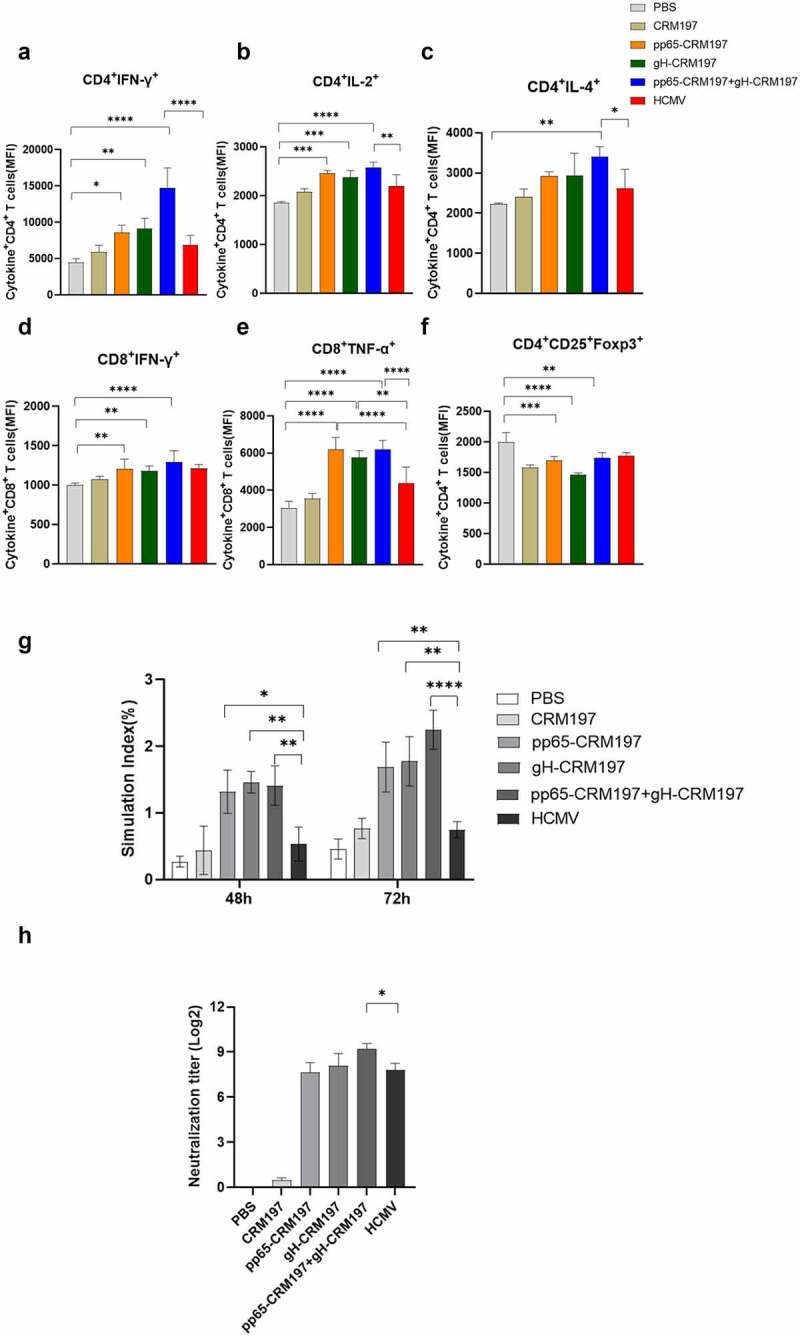
Evaluation of immune responses in three peptides-CRM197 vaccines. On the 35th day after the first immunization, the lymphocyte single-cell suspension was obtained, and the expression of cytokines was analysed by flow cytometry. The MFI expression level of IFN-γ (a), IL-2 (b), and IL-4 (c) in CD3+CD4+T cells, IFN-γ (d), and TNF-α (e) in CD3+CD8+T cells, and Foxp3 (f) in Treg cells. The inactivated HCMV was used to stimulate splenic lymphocytes of mice, and lymphocyte proliferation was detected at 48 and 72 h (g). Detection of neutralizing antibody titre in mice serum (h). All data were expressed as mean ± SD, * means *P* < .05, ** means *P* < .01, *** means *P* < .001, and **** means *P* < .0001 (one-way ANOVA).

To evaluate the immunogenicity of three peptide – CRM197 vaccines, the mice were immunized with OVA, CRM197, pp65-CRM197, gH-CRM197, pp65-CRM197+gH-CRM197, and inactivated HCMV. The mice were sacrificed to collect spleens and serum for further research after three immunizations for 7 days. To measure the level of cellular immunity, we examined the IFN-γ, IL-2 (Th1 cytokines), and IL-4 (Th2 cytokines) expressing in CD4+T cells, IFN-γ, TNF-α expressing in CD8+T cells. As shown in Figures 5a-e and S2, compared with the OVA group, the expression of cytokines in three peptide-CRM197 vaccine groups increased. The expression of IFN-γ and TNF-α in CD8+T cells was significantly increased in all peptide – CRM197 vaccine treated groups which was higher than that in the inactivate HCMV treated group, especially the pp65-CRM197+gH-CRM197 group. Moreover, compared with the PBS group, the expression of IFN-γ, IL-2, and IL-4 in CD4+T cells was significantly upregulated in all peptide-CRM197 vaccines treated groups, especially the expression of IFN-γ and IL-2. In addition, the expression of IFN-γ in the pp65-CRM197+gH-CRM197 group showed 3.3-, and 2.14-fold increase compared with that in the PBS and inactivated HCMV groups, respectively. These results indicated that three peptide – CRM197 vaccines induced immunity is dominated by Th1 cell immunity and the effect is better than that of the inactivated HCMV treated group. We also detected Foxp3 expressing in Treg cells, which functions as an immunosuppressive factor. The results indicated that the expression of Foxp3 reduced in all peptide – CRM197 vaccine treated groups (Figure 5f), indicating that three peptide-CRM197 vaccines could promote innate and adaptive immune responses via multiple synergistic effects. We also measured the effect of three peptide – CRM197 vaccines on the proliferation of mice spleen lymphocytes. We treated immunized mice spleen lymphocytes with inactivated HCMV and observed that the proliferation of lymphocytes in all peptides – CRM197 vaccines groups was significantly higher than that in the inactivated HCMV group (Figure 5g). The neutralizing antibodies produced by the peptides – CRM197 vaccines reached a 2^8^ titre, especially the pp65-CRM197+gH-CRM197 group produced a higher level of antibodies than the inactivated HCMV group (Figure 5h). The above results showed that three peptide-CRM197 vaccines could induce effective immune responses, including brilliant cellular and humoral immune response.

## Discussion

Natural infection of HCMV does not expose the dominant antigenic epitopes efficiently, inactivated and attenuated HCMV vaccines cannot provide effective protection, and have shown specific safety issues [38]. Nevertheless, T and B cell epitope vaccines have been used to develop HCMV vaccines, and have shown remarkable results [23,39]. Therefore, we used bioinformatics to screen the dominant epitope regions of T and B cells in pp65 and gH, so as to produce more effective immune responses. Compared with the complete pathogen, the predicted peptides are easy to synthesize, induce fewer allergic reactions and also expose effective antigenic epitopes better [40]. Bioinformatics has been used to predict the characteristics of the virus and epitopes present in the pathogen, which has dramatically accelerated the development of the vaccine [41]. Therefore, bioinformatics is an excellent approach to predict T and B cell epitopes. In this study, we predicted a total of ten T cell epitopes and five B cell epitopes of pp65 (Table 1) and nine T cell epitopes and five B cell epitopes of gH (Table 2). Two epitopes with the highest degree of overlap and score were screened to construct the peptide vaccine. As a protein carrier the peptides – CRM197 conjugation could maximize the maintenance of the dominant antigenic epitopes, increase the size of the polypeptides, and prevent premature elimination by the body, so as to be fully exposed to the immune system [42]. Thus, we conjugated the synthesized polypeptides to CRM197, which increased the antigenic exposure as well as immunogenicity of the vaccine to activate T cells (Figure 1a-d).

A critical point in the immunization strategy is to ensure that the vaccines are recognized and ingested effectively by APCs, like DCs [43]. The activation of DCs showed that the expression of MHCII, CD80, CD86, and CD40 increased in all peptide – CRM197 vaccines groups (Figure 2a-d and 3a-b), this finding was consistent with that of other HCV recombinant peptide vaccines [44,45]. Three peptide-CRM197 vaccines also increased the production of proinflammatory factors (TNF-α, IL-6, IL-1β, and IL-12p70) in DCs (Figures 2e-h), which subsequently induced T cells activation [45]. In addition, the upregulation of surface markers and inflammatory cytokines of DCs in the peptide-CRM197 vaccine groups was higher than that of the positive control group (inactivated HCMV and LPS group), especially in the pp65-CRM197+gH-CRM197 group. This finding suggested that using bioinformatics to predict the dominant antigenic epitope is feasible for better antigen presentation of DCs, and the effect is better when the two peptides are used together. Cao, *et al*. reported that HCMV infection inhibits the maturation of DCs and impairs cell function, resulting in reduced proliferation and cytotoxicity of DCs-specific T cells [46]. In contrast, the peptide-CRM197 vaccine could avoid damaging the cellular function of DCs and present antigens better, guaranteeing prominent immune effects. Since antigens could activate APCs through the p38 MAPK signalling pathway to induce cytokine production [45,47], we correspondingly detected the phosphorylation of essential proteins in the p38 MAPK signalling pathway in DCs, which significantly enhanced after treatment with three peptide-CRM197 vaccines (Figures 4a-b). When pretreatment with the inhibitor of p38 MAPK, the expression of CD80, CD86, CD40, and MHCII decreased as expected (Figure 4c), further proving that three peptides-CRM197 vaccines activated DCs via the p38 MAPK signalling pathway. TLR4 could recognize specific viral proteins on the cell surface, leading to the activation of downstream p38 MAPK pathway [48,49]. Therefore, the three peptide vaccines constructed in this study might be recognized by TLR4 and subsequently stimulate the downstream p38 MAPK pathway to activate DCs.

Because of the species specificity of HCMV, there is no suitable animal model at present [50], so we evaluated the immunogenicity of three peptide-CRM197 vaccines based on the immune level of mice. Since humoral immunity alone could not control HCMV infection, so we focused more on improving cellular immune responses. Pathogen-associated specific CD8+T cells, which produce high levels of IL-2, IFN-γ, TNF-α, perforin, and granzyme B to regulate the infection, are essential for HCMV immunity [51–53]. In this study, three peptide-CRM197 vaccines promoted the proliferation of mice spleen lymphocytes (Figure 5g), and effectively activated CD8+T cells (Figures 5d-e). In addition, three peptide-CRM197 vaccines also induced higher expression of IFN-γ and IL-2 in CD4+ T cells (Figure 5a-b), indicating that three peptides-CRM197 vaccines induced Th1 dominant responses. Studies have also shown that Th1 cells are necessary to control cytomegalovirus infection in immunocompromised transplant recipients and HIV-infected patients, which could directly inhibit viral replication by secreting IFN-γ [54,55]. Furthermore, decreased expression of Foxp3 was observed in Treg cells (Figure 5f) of three peptides-CRM197 vaccines groups, which could avoid immunosuppressive response and further enhance inflammatory response [56]. Meanwhile, the peptides-CRM197 vaccines induced effective neutralizing antibodies of a 2^8^ titre (Figure 5h). These results revealed that three peptides – CRM197 vaccines not only stimulated a high level of cellular immunity but also neutralized antibodies, demonstrating that the peptide-CRM197 vaccines could protect body through the synergistic effect of cellular and humoral immunity. Compared with the use of a single vaccine, combination of two peptide vaccines could induce higher immune responses, the trend was same as the activation of DCs. Therefore, combination of two peptide vaccines should be used in future research to obtain more efficient immunity. However, there were still several limitations in our experiments. Due to the high specificity of HCMV infection [50], there is no suitable animal model to conduct challenging experiments. Furthermore, our future research would verify the immunogenicity of the peptide – CRM197 vaccine on human cells.

**Figure 6.**
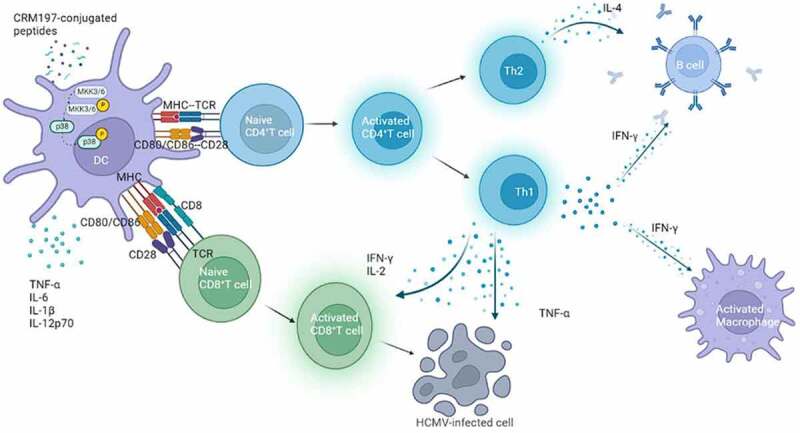
Schematic diagram of the peptide-CRM197 vaccines activating immune responses.

In conclusion, we successfully screened effective T and B cell epitopes using bioinformatics software and constructed three peptide – CRM197 vaccines. Three peptide-CRM197 vaccines could activate DCs through the p38 MAPK signalling pathway *in vitro* and *in vivo* as well as induce effective humoral and strong cellular immunity to provide excellent immune effects by the synergistic effect (Figure 6). Therefore, our study provided a novel method for the development of HCMV peptide epitope vaccines based on pp65 and gH.

## Data Availability

The authors confirm that the data supporting the findings of this study are available within the article [and/or] its supplementary materials
